# Benchmarking a new radiochromic film type for photon and proton beam dosimetry

**DOI:** 10.1016/j.phro.2026.100985

**Published:** 2026-05-05

**Authors:** Peter Kuess, Barbara Knäusl, Sarah Helletzgruber, Dietmar Georg, Hugo Palmans, Wolfgang Lechner

**Affiliations:** a Department of Radiation Oncology, Medical University of Vienna, Währinger Gürtel 18-20, Vienna, A-1090, Austria; b MedAustron Ion Therapy Center, Marie Curie-Straße 5, Wiener Neustadt, A-2700, Austria; c Christian Doppler Laboratory for Image and Knowledge Driven Precision Radiation Oncology, Medical University of Vienna, Währinger Gürtel 18-20, Vienna, A-1090, Austria; d Radiotherapy and Radiation Dosimetry, National Physical Laboratory, Hampton Rd, Teddington, TW 11 0LW, United Kingdom

**Keywords:** Film dosimetry, EBT4 radiochromic films, Proton dosimetry, Film storage

## Abstract

**Background and Purpose:**

EBT4 Radiochromic films (RCFs) are replacing the well-described EBT3 type. This study aimed to benchmark the EBT4 type against its predecessor for photon (kV and MV) and proton beam dosimetry.

**Materials and Methods:**

Differences between the film types regarding the signal-to-noise ratio (SNR) were investigated in 6 MV photon and 148.2 MeV proton beams in the dose range of 0.2 Gy to 10 Gy for all RGB channels. The effect of the dose-averaged linear energy transfer (LET${}_{d}$) on the response of both film types was compared within a proton spread-out Bragg peak (SOBP). Furthermore, the relative response in low-kV X-rays was studied and the effect of long-term storage on both film types.

**Results:**

The SNR of EBT4 films was statistically superior for MV-photon irradiations above 1.5 Gy in the red channel by up to 29 %. For low-dose proton irradiations, the SNR of EBT3 was higher. The underresponse of both film types in a proton SOBP reached 5 to 7 %. In low kV X-ray beams, EBT4 deviated from EBT3 on average by 2.5 %. For irradiations of films with a dose of 2 Gy or less after 216 days of storage, smaller differences in terms of pixel values were observed for films placed at 10°C.

**Conclusions:**

The presented results are in accordance with recent studies on EBT4 films and extend the knowledge on this film type, especially regarding proton beams and film storage.

## Highlights

• Similar linear energy transfer dependency of EBT3 and EBT4 in clinical proton beams.
• Photon beams: EBT4 films showed superior signal-to-noise ratio of up to 29%.
• Proton beams: Superior signal-to-noise ratio of EBT4 only for doses ≥ 1.50 Gy.
• Pixel value stability <1% at 10 °C storage temperature over 216 days.
• Pixel value change of films stored at room temperature reached up to 2.4%.

## Graphical abstract

**Figure:**
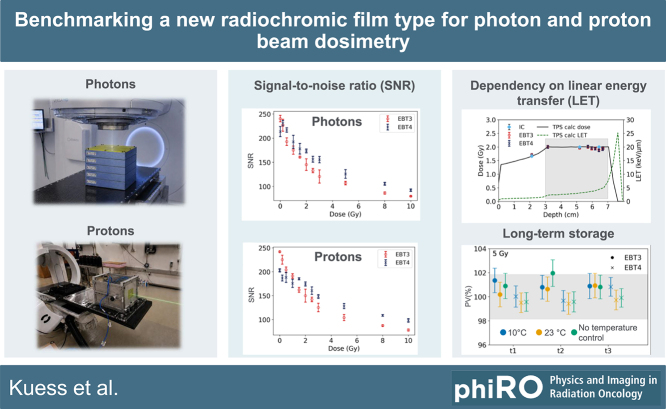

## 1 Introduction

Highly reliable dosimetric equipment is the backbone for beam characterization, commissioning, quality assurance, and research in radiation oncology [1]. One important tool is the Radiochromic film (RCF), which provides comprehensive information on the 2D dose distribution with high resolution [2].

The first EBT RCF was launched in 2004 [3], [4] and has been continuously developed since. In 2011, the EBT3 type was introduced, which provides a symmetrical structure on both sides of the sensitive layer [5], [6]. Since 2022, this type is being replaced by the EBT4, having identical structure and layer thicknesses as its predecessor.

The notable difference between EBT3 and EBT4 is the changed active fluid of the films [7], which is expected to result in an improved signal-to-noise ratio (SNR) of EBT4 films compared to EBT3 films. This was recently confirmed for photon beams in two studies [7], [8]. Other studies investigated different performance aspects of EBT4 RCFs, namely the effects of scanning orientation and the lateral response artifact [9], the energy dependence for kV and MV photon beams [10], as well as post-irradiation darkening effects [11].

Recently, EBT4 RCFs have also been reported in the context of total body irradiation [12], dose verification in LINAC-based stereotactic radiosurgery treatments [13], [14], investigations of dosimetric effects of a silicon-based gel [15], and ultra-high-dose dosimetry [16]. The published literature on EBT4 dosimetry in proton beams is currently scarce [17], [18]. However, RCFs are also used in particle beams but are affected by the increased dose-averaged linear energy transfer (LET${}_{d}$) of the ion beams. The LET${}_{d}$, which quantifies the energy deposited per unit path length of a charged particle, can lead to an underresponse of RCFs through two primary mechanisms. First, radical recombination can occur when densely generated free radicals recombine before initiating polymerization. Second, saturation of polymerization arises when the number of ionizations exceeds the available monomers, limiting further signal generation [19].

This study aimed to benchmark the EBT4 film type against the well-described EBT3 type for clinical proton and 6 MV photon beams. Furthermore, EBT3 and EBT4 RCFs were stored at three different conditions for 216 days and irradiated at three post-calibration time points to assess how storage conditions and time affect the pixel value (PV) response.

## 2 Materials and methods

### 2.1 General settings

During all experiments, EBT4 RCFs (lot#7192301) were used together with EBT3 films (lot#11022201) (Ashland Specialty Ingredients, G.P., Bridgewater, NJ, USA). The active monolayers of both types have a thickness of 28 µm, coated with two symmetric layers of 125 µm matte-polyester. Handling of the RCFs was in accordance with international recommendations [2], [20] and previous work [21]. All film sheets used in this study were cut into pieces of 4 × 5 cm^2^ with scissors.

An EPSON Expression 12000-XL flatbed scanner (Seiko Epson Corporation, Nagano, Japan) was used to digitize the RCFs in combination with Epson Scan 2 software (v6.6.45). The films were placed at the center of the scanner, with the short edge of the film oriented perpendicular to the scanning direction. All film scans were acquired in 48-bits RGB channel transmission mode, with all correction options turned off at 150 dpi, 48 h post irradiation. The response of each film was averaged over three consecutive scans using the extracted scalar values from each scan. For proton and MV beams, the mean PV and its corresponding standard deviation were obtained from the central 2 × 2 cm^2^ area of each scan. Due to increased beam heterogeneity, a smaller area of 1 × 1 cm^2^ was evaluated for the RCFs irradiated with kV X-rays.

Data evaluation was performed using in-house Python scripts (Python 3.9, NumPy, SciPy, Matplotlib, Pandas). Uncertainty estimations are presented in the Supplemental Table S4 and S5 and are based on Lechner & Palmans [22].

### 2.2 Irradiation setup

Irradiation with 6 MV photon beams was executed at an Elekta Versa HD Linac (Elekta, Stockholm, Sweden), employing a 10 × 10 cm^2^ field. Film sheets were placed within a Gammex solid water phantom (Sun Nuclear, Melbourne, USA) at a source-to-surface distance (SSD) of 90 cm. 15 cm phantom material was placed below the films and 10 cm above. Monitor units (MUs) to dose calibration was performed with a PTW 30006 Farmer ionization chamber (PTW, Freiburg, Germany) in the Gammex phantom. For film calibration, ten dose levels, in accordance with the vendor specifications, were irradiated four times (0, 0.2, 0.5, 1, 1.5, 2, 3, 5, 8 and 10 Gy). In each measurement, one EBT3 and one EBT4 film were irradiated in a stacked configuration, alternating the top film.

Proton irradiations were performed at a synchrotron-based ion beam therapy facility (MedAustron, Wiener Neustadt, Austria) employing a horizontal beamline [23], [24]. The nominal proton beam energy was 148.2 MeV, corresponding to a range of 150.4 mm in water. A 10 × 10 cm^2^ field was irradiated with a spot spacing of 0.2 cm in horizontal and vertical directions. The films were positioned at a physical depth of 19 mm RW3 (PTW), corresponding to 19.8 mm water equivalent thickness (WET). They were arranged in a stack of four, alternating both film types. For backscatter, 40 mm RW3 was placed behind the films additionally. All films were irradiated at the identical dose levels as for MV photon beams. Dose measurements at the film position, corresponding to the chamber’s reference point, were performed with a calibrated PTW 34001 Roos chamber. The WET of EBT4 RCFs was determined by relative range measurements with a PTW Peakfinder and three stacked films [21], [25], [26].

RCFs were further positioned in a proton spread-out Bragg peak (SOBP) with 4 cm modulation and a depth of 7 cm. The field size was 12 × 12 cm^2^ with a constant spot spacing of 0.2 cm and energies ranging between 63.5 to 100.4 MeV. Plans were generated using the treatment planning system (TPS) RayStation 2024 A (v15.1.100.0) with the Monte Carlo dose engine v5.6 (RaySearch Laboratories, Stockholm, Sweden), which also enables LET${}_{d}$ calculation in water from primary and secondary protons [27]. At various positions (i.e. WET=2.13, 3.17, 5.20, 5.74, 5.97, 6.21, 6.44, 6.68 and 6.71 cm) along the SOBP both film types were placed next to each other on RW3 plates. The measurements were performed twice with a planned dose of 2 Gy in the SOBP. At three positions (i.e. WET=2.12, 5.19 and 6.43 cm), the dose was measured with a PTW 34001 Roos chamber.

To relatively compare the response behavior in kV X-rays, both film types were irradiated with a commercial X-ray unit (YXLON GmbH, Hamburg, Germany) with a filtration of 3 mm Be [28]. As described in et al.Khachonkham, a stack of two films was irradiated at 15, 20, 25, 30, 50, 80 and 100 kV with 1 to 2 Gy [21]. The corresponding half-value layers (HVLs) were 0.050, 0.060, 0.068, 0.075, 0.080 and 0.084 mm Al. Each irradiation was repeated three times. To account for attenuation effects, the films’ positions were changed after 50 % of the irradiation time. Thus, both films received the same dose. The response of EBT4 films was normalized to that of EBT3.

### 2.3 Film characteristics and analysis

PVs were converted into dose applying a 4th-order polynomial fit. Background scans of each film were acquired prior to irradiation and incorporated into the calibration by normalizing the PVs of the irradiated scans to the corresponding background values. Sensitivity curves were calculated following Eq. (1).

(1) $$\frac{\delta PV}{\delta D}=\frac{PV_{2}-PV_{1}}{D_{2}-D_{1}}$$

All RGB channels were investigated separately and combined using the triple-channel correction [29]. While single-channel correction is a straightforward approach converting the PV of a film read-out to dose, triple-channel correction employs all available color channels and an additional optimization step. Since all channels were exposed to the same amount of radiation, the optimization process varies the thickness of the active layer to maximize the consistency between the dose reported by the three channels. The output of the triple-channel method is a dose map and a map reflecting the relative thickness of the active layer [29].

SNR was evaluated as the ratio of the mean PV to its standard deviation, computed for each RGB channel at each dose level. The SNR was averaged from each of the four film pieces obtained from different film sheets. Furthermore, SNR was determined for the reported dose, based on single RGB and triple-channel evaluation. A Wilcoxon Rank-Sum test was applied to test for a significant difference in SNR between the film types.

The effect of storage conditions on the performance of RCFs was evaluated at four dose levels (0, 0.5, 2 and 5 Gy) for both film types using a 6 MV photon beam. Storage was carried out under three different conditions: at 10 °C and at 23 °C, both temperature controlled with fluctuations of up to 2 °C, and in a non-temperature-controlled place which exhibited variations between 16.8 °C and 30.1 °C during the study period (see Figure S5 for detailed temperature curve). The films were placed in storage locations after the calibration was performed and film response was subsequently acquired at three later time points (20, 127 and 216 days). Results are presented as PV normalized to the calibration PV (day 0) for each film type and dose.

## 3 Results

The behavior of PV as a function of dose for RGB channels comparing EBT3 and EBT4 films for photon and mono-energetic proton irradiation (Fig. 1a and c) showed a smilar trend among both beam qualities. The coefficient of determination was close to unity for all RGB channels in proton and photon beams. Fig. 1b and 1d depict the corresponding sensitivity curves according to Eq. (1). None of the film types showed any response variation between these photon and proton reference beam qualities. The results indicate that a green channel evaluation for higher dose regions was slightly less advantageous for EBT4 than for the EBT3 film type. The relative PTW Peakfinder measurements within a proton beam resulted in 360 µm WET for a single EBT4 film sheet.

As shown in Table 1 for doses above 1.5 Gy, EBT4 generally exhibited a higher SNR expressed as scanned PV than EBT3, with relative differences reaching up to 29 % for the red and green channel and 14 % in the blue channel (PVs for the green and blue channels are listed in the Supplementary Table S2. At doses ≤ 1.5 Gy the SNR of proton irradiated EBT4 films was inferior to the SNR of EBT3 in all RGB channels. This was contrary to photon irradiations, where the EBT4 SNRs were similar or higher for doses of 0.2 to 1.5 Gy, ranging between -3 to 11 %, depending on the RGB channel. The difference of SNR expressed as scanned PV (red channel) was statistically significant (p<0.05) for photon and proton irradiations at doses ≥ 1.5 Gy in favor of EBT4 films.Fig. 1Left column: dose response curves in PV for EBT3 and EBT4 films in RGB channels (a) irradiated with a 6 MV photon and (c) with a mono-energetic 148.2 MeV proton beam. Right column: sensitivity curves for EBT3 and EBT4 films for RGB channels; (b) 6 MV photons and (d) 148.2 MeV protons. Solid lines correspond to EBT3 films, whereas dashed lines indicate EBT4 films.Fig. 1

**(a):**
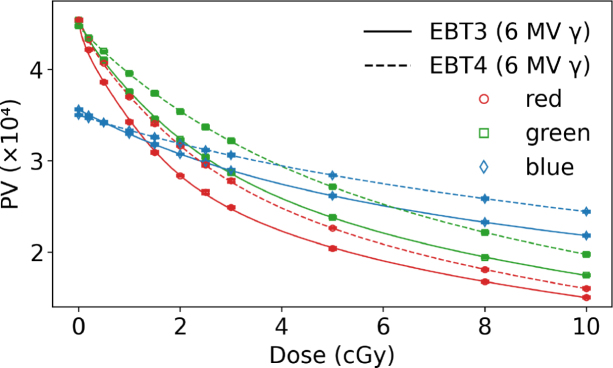
.

**(b):**
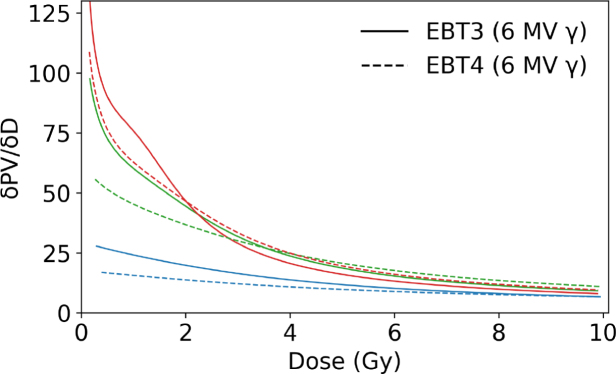
.

**(c):**
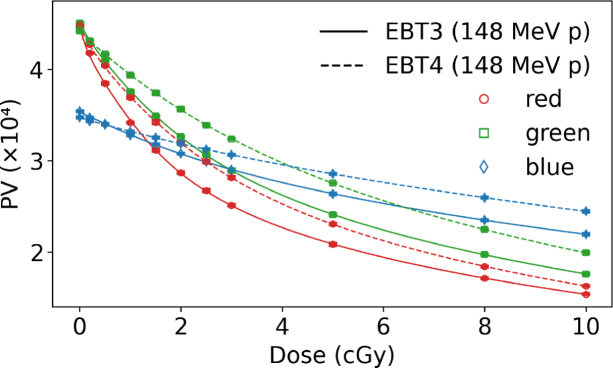
.

**(d):**
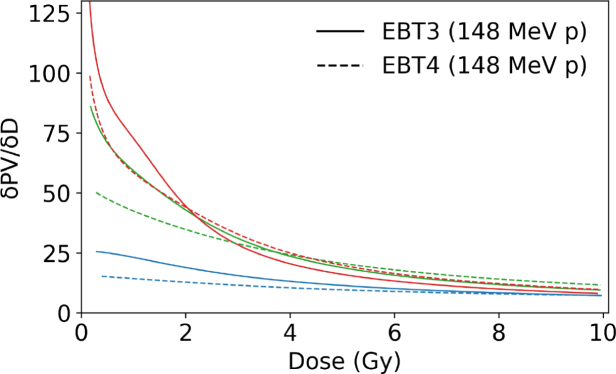
.

A similar trend was observed for the SNR of the reported dose, where EBT4 exceeded EBT3 by up to 34 % for photons and 51 % for protons using the red channel (Table 1) at doses above 1.5 Gy. Notably, when using the triple-channel correction method, the SNR differences for doses ≥ 5 Gy ranged from 70 to 99 % (Table S3). For all dose levels below 2 Gy EBT3 films exhibit higher SNR values than EBT4, independent of the applied calibration method. Note, that in general the SNR reported as dose was only significantly different for higher dose levels (≥3Gy).

Within a proton SOBP, the PTW 34001 Roos chamber measurements agreed with the TPS calculated dose within 0.5 %, as depicted in Fig. 2. Table S4 presents the uncertainty estimation. Note that the TPS beam model is derived from measurements traceable to the same primary standard used in this work. The film measurements in the entry region (i.e. 2.1 cm) agreed with the TPS calculated dose for both film types within 3 % for red- and triple-channel. The green and blue channels showed larger discrepancies of up to 5.6 % and 12.5 %, respectively in comparison to the TPS calculated dose. For all calibration methods (except blue channel) the deviation between the dose measured with films and calculated by the TPS in the entrance region was smaller for EBT4 films. Starting mid-SOBP, the response of both film types decreased, with LET${}_{d}$ values above 2 keV µm^-1^. For red- and triple-channel, the deviations for EBT3 and EBT4 reached up to 5 to 7 %. For the green channel, deviations were below 3 % for both film types. Note, that the largest deviations were not measured at the position with the highest LET${}_{d}$ (i.e. 4.95 keV µm^-1^) but at positions with LET${}_{d}$ values ranging between 4.0 to 4.4 keV µm^-1^, depending on the RGB channel.

**Table 1:**
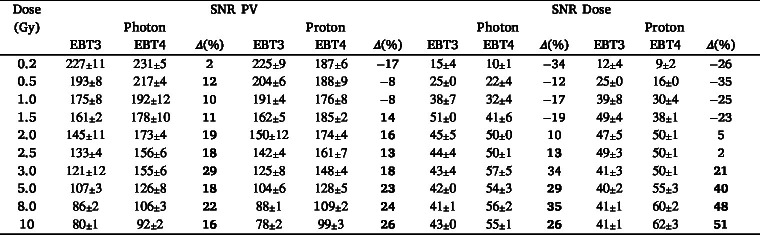
SNR expressed as scanned PVs and reported dose in comparison between EBT3 and EBT4 films at different dose levels for the red channel for photon and proton beams. Values in round brackets indicate the percentage difference between film types (EBT4 relative to EBT3). Bold values imply statistically significant differences (p<0.05), with EBT4 performing significantly better than EBT3.

**Fig. 3:**
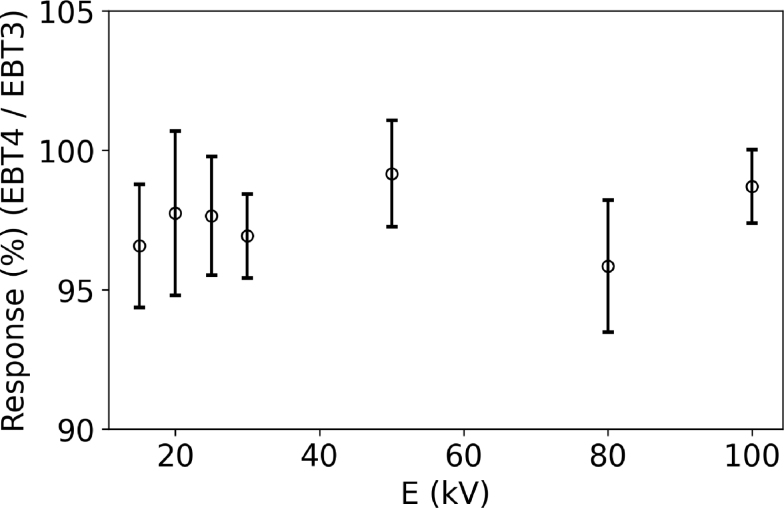
Response ratio of EBT4 to EBT3 for energies between 15 to 100 kV. For each energy, three film pieces were irradiated per film type.

Measurements in low-kV X-ray beams showed a reduced response of EBT4 films for energies between 15 to 100 kV compared to EBT3 (Fig. 3). The average deviation between the two film types (red channel) was 2.5 %, with maximum 4.2 % at 80 kV for all investigated energies. Triple-channel evaluation yielded similar values, with an average deviation of 2.3 % and a maximum of 4.5 %. The green and blue channels showed slightly higher deviations, with averages of 3.8 % and 4.1 % and maxima of 5.9 % and 5.6 %, respectively.Fig. 2Response of both film types in a proton SOBP with a modulation width of 4 cm at a depth of 7 cm, evaluated using the red channel. (a) depicts all measurement points over the full range and (b) focuses solely on the SOBP region. The gray-shaded area in (a) indicates the target region. Film measurements (EBT3 = red circle, EBT4 = dark blue cross) represent the mean of two repetitions. The black solid line shows the TPS-calculated dose, while the green dashed line corresponds to the TPS-calculated LET${}_{d}$. At three positions, the dose was additionally measured using a PTW 34001 Roos chamber (light blue diamonds).Fig. 2

**(a):**
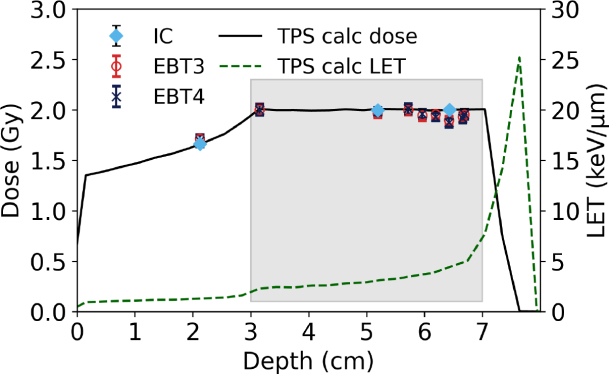
.

**(b):**
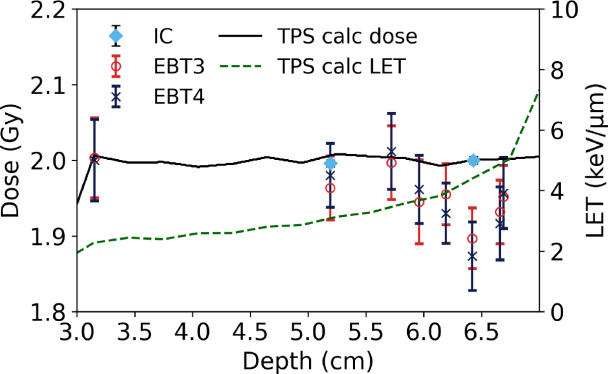
.

**Fig. 4:**
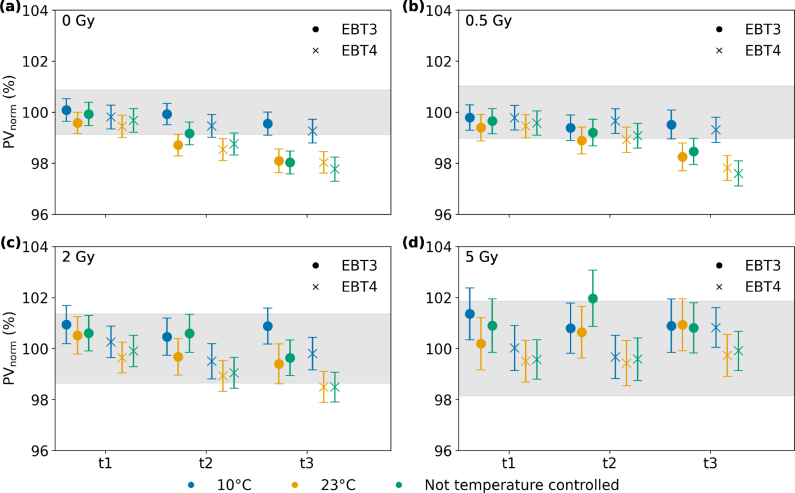
PV normalized to the value at calibration over time. EBT3 films are illustrated with circles and EBT4 films with crosses. The colors represent different storage conditions (10°C; 23°C; not temperature controlled). The evaluation was performed for four different dose levels; 0 Gy (a), 0.5 Gy (b), 2 Gy (c), and 5 Gy (d). The gray shaded area indicates the 2-sigma interval, averaged over all films at the given dose level. The time points t1-t3 correspond to 20, 127 and 216 days after film calibration.

For non-irradiated films (Fig. 4a) and those irradiated with 0.5 Gy (Fig. 4b), a clear decline was observed in the red channel for both film types when stored at 23°C or under non-temperature-controlled conditions, with changes up to 2.4 % (0.1 Gy). At 0.5 Gy the PV change at the third time point was more pronounced for EBT4 films. At these dose levels (i.e. 0 Gy and 0.5 Gy), only minor changes were found for films stored at 10°C with a maximum decline of 0.7 % (0.03 Gy) for EBT4. For 2 Gy and 5 Gy (Fig. 4c,d) the effect diminished while the PV uncertainty increased. The green channel evaluation showed basically the same qualitative behavior. Uncertainty estimations for film storage measurements are presented in Table S5. An overview and comparison between the film types for all results obtained during this study is summarized in Table 2.

Investigations on film darkening are shown in the Supplementary Figure S1–S3 and elaborated in the Supplementary Material A. Inter-scan variability of EBT3 and EBT4 RCFs, as proposed by Lewis & Devic [30], is shown in the Supplementary Figure S4 and outlined in the Supplementary Material B.

**Table 2:** Overview comparison of key characteristics of EBT3 and EBT4 RCFs.

Characteristic	Observation
SNR (photon)	EBT4 > EBT3
SNR (proton)	EBT4 > EBT3 for doses >2 Gy
Proton LET dependency	Similar underresponse with increasing LET
Water equivalent thickness	Both 360 µm
kV photon response	Slightly lower response of EBT4 (∼2.5%)
Film storage	Similar behavior under all tested conditions

## 4 Discussion

This study characterized the EBT4 film type against its predecessor EBT3 in MV- and kV-photon beams as well as proton beams. The claimed improved SNR of the EBT4 film could be confirmed for dose levels above 1.5 Gy. However, at lower dose regions this effect was not observed in clinical proton beams. Among proton and kV irradiations, the behavior of EBT4 and EBT3 films were very similar.

EBT4 films showed systematically higher SNRs, confirming previous findings [7], [8]. In et al.Palmer, the SNR of EBT4 increased from 10 to 23 % in the red and green channels for scanned PVs and up to 60 to 75 % at 10 Gy when expressed as reported dose [7]. Also et al.Shameem reported a clearly higher SNR of EBT4 for all investigated dose levels (i.e. 0 to 100 Gy) in the red channel compared to EBT3 [8]. The present study also confirmed the large difference when SNR is expressed as dose, which reached nearly 100 % when applying the triple-channel correction method. While et al.Palmer did not confirm that the difference in SNR between EBT3 and EBT4 was statistically significant, we can report *p*-values of <0.05 for SNR as PV for photon and proton irradiations in the red channel for doses higher than 1.5 Gy. The films irradiated with kV beams could also be used to calculate the SNR expressed as scanned PV. For a reported dose between 1 to 2 Gy the results were in agreement with MV photon irradiations as presented in Table 1.

As this is the first investigation of EBT4 films in a proton beam, further studies are encouraged to examine the SNR in low-dose proton beams in more detail. Our experiments were performed at an active scanning facility, which could potentially differ from a passive scattering delivery technique. For further characterization in the proton beam, the WET of an EBT4 RCF was also measured. The determined 360 µm WET is identical to the value obtained for EBT3 films by et al.Khachonkham [21].

Also within a proton SOBP the EBT4 film showed very similar behavior to EBT3 film. Results obtained using the red channel were comparable with et al.Khachonkham, where an under-response of 6.1 % was reported for EBT3 films [21]. This is also in agreement with et al.Anderson where an underresponse of EBT3 films up to 10 % for LET${}_{d}$ values below 5 keV µm^-1^ was reported [31]. Note that in our study, the triple-channel correction was very similar to the red channel; however, the green channel seemed less affected by LET${}_{d}$ quenching. Whether this observation holds for different film batches and across a broader LET range requires further investigation.

RCFs are energy independent in MV photon beams [6], [10], but show an energy dependence in kV beams [10], [32], [33]. In this study, no dedicated kV calibration was performed, but the response of the new film type was put in relation to EBT3 films.

Studies on ideal conditions during film storage are scarce. According to the vendor, RCFs can be stored at room temperature (20 to 25°C), although storage at refrigerator temperature is recommended. In this study, we investigated the response behavior within these recommended temperatures. et al.Trivedi studied the impact of short-term storage (≤48h) at 2, 20 and 40 °C and found that storage at 40 °C resulted in an overestimation of the dose by 7 % [34]. This is partly in accordance with our findings, where the PVs of films stored outside the refrigerator were generally lower, indicating an enhanced darkening. However, et al.Trivedi focused on short-term storage, while our study investigated storage times up to 216 days. Our results indicate RCFs placed at 10°C are more stable over time. Baseline changes associated with storage can, to some extent, be corrected by using background scans acquired prior to irradiation. However, potential changes in the sensitivity of the films cannot be restored that way.

In conclusion, the EBT4 RCF demonstrates an improved SNR with dose- and channel-dependent behavior, although no improvement was observed in the low-dose proton region. It can be used in proton beams under similar restrictions as the EBT3 films (i.e. under-response in higher LET${}_{d}$ regions). The storage study indicates that both film types are more affected when stored at room temperature than at 10°C.

## CRediT authorship contribution statement

**Peter Kuess:** Writing – original draft, Visualization, Software, Methodology, Investigation, Data curation, Conceptualization. **Barbara Knäusl:** Writing – review & editing, Methodology, Investigation, Funding acquisition. **Sarah Helletzgruber:** Validation, Software, Data curation. **Dietmar Georg:** Writing – review & editing, Resources, Methodology. **Hugo Palmans:** Writing – review & editing, Methodology, Investigation, Conceptualization. **Wolfgang Lechner:** Writing – review & editing, Visualization, Validation, Software, Methodology, Investigation, Conceptualization.

## Declaration of competing interest

The authors declare that they have no known competing financial interests or personal relationships that could have appeared to influence the work reported in this paper.

Given her role as Editor-in-Chief, Barbara Knäusl had no involvement in the peer-review of this article and had no access to information regarding its peer-review. Full responsibility for the editorial process for this article was delegated to another journal editor.

## Appendix A Supplementary data

MMC S1 In the Supplementary Material A post-irradiation growth of the optical density is shown in comparison for EBT3 and EBT4 radiochromic films. Supplementary Material B provides information on the scan-to-scan variability of EBT3 and EBT4 films. Table S2 and S3 provide additional information to the signal-to-noise ratio. Table S4 and S5 list the uncertainty budget of reference measurements and film storage measurements. MMC S1
