# Supplementary material for: Benchmarking a new radiochromic film type for photon and proton beam dosimetry
Source: Phys Imaging Radiat Oncol. 2026 May 5;39:100985. doi: 10.1016/j.phro.2026.100985 (PMC13156622; doi:10.1016/j.phro.2026.100985)
Supplement: MMC S1 — In the Supplementary Material A post-irradiation growth of the optical density is shown in comparison for EBT3 and EBT4 radiochromic films. Supplementary Material B provides information on the scan-to-scan variability of EBT3 and EBT4 films. Table S2 and S3 provide additional information to the signal-to-noise ratio. Table S4 and S5 list the uncertainty budget of reference measurements and film storage measurements. [file mmc1.pdf]

Supplementary Material

Supplementary Material A

Radiochromic films (RCFs) were scanned periodically for 45 h post irradiation. Four films (two EBT3, two EBT4) were scanned simultaneously; for each film type one film was irradiated with 2 Gy (6 MV photons) while the other served as background. Scans were taken every 15 min during the first 6 h, every 30 min from 6 to 12 h, and hourly thereafter. Each scan was repeated three times, and the averaged image was used to calculate the net optical density (OD_{net}) (Equation S1) and its change relative to the value at 0.5 h post-irradiation. PV_{Bgr} and PV_{Irr} denote the pixel values (PVs) of the background scan and the irradiated scan, respectively.

$$OD_{\text{net}} = \log_{10}\left(\frac{PV_{\text{Bgr}}}{PV_{\text{Irr}}}\right) \tag{S1}$$

The change of OD_{net} with time for the red channel was fitted using the bi-exponential function proposed by Khan *et al.* (Equation S2) [1].

$$OD_n(t) = OD_{n,\infty} - C_1 \exp\left(-\frac{t}{T_1}\right) - C_2 \exp\left(-\frac{t}{T_2}\right), \tag{S2}$$

As shown in Figure S1 for the red channel, the OD_{net} increased by 4.0 % and 5.6 % within the first 6 h after irradiation with 2 Gy (6 MV photons) for EBT3 and EBT4 films, respectively. Between 6 h and 24 h, the increase was 2.7 % for EBT3 and 3.7 % for EBT4, while between 24 h and 44 h the increase was 1.2 % (EBT3) and 1.6 % (EBT4). After 35 h the increase was below 0.4 % and 0.5 % for EBT3 and EBT4 films, respectively. Comparable results were obtained for the green and blue channels. In contrast, Figure S2 shows the film darkening as change of pixel value for both film types for non-irradiated films and films irradiated with 2 Gy.

The bi-exponential fit function (Equation S2) showed very good agreement with our experimental data, yielding coefficients of determination of $R^2 = 0.999$ for EBT4 and $R^2 = 0.997$ for EBT3 (Figure S3). Table S1 provides the respective model parameters.

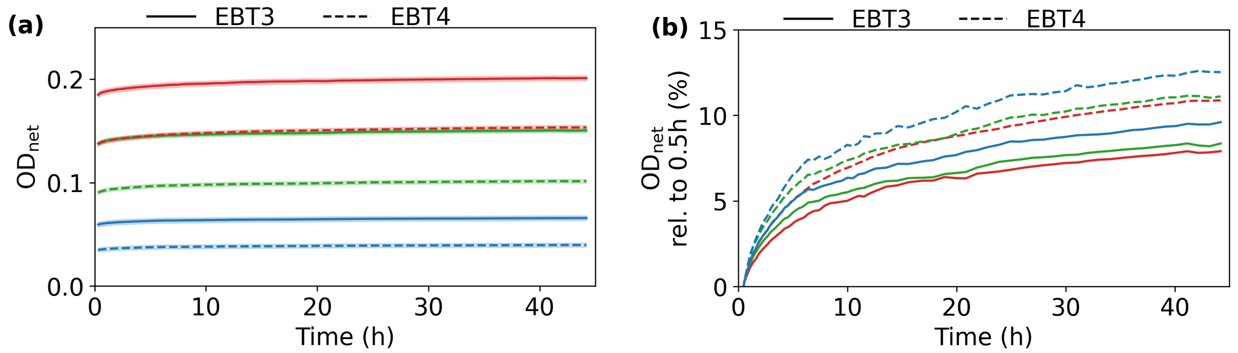

Figure S1: (a): Post-irradiation growth of the net optical density for EBT3 (solid lines) and EBT4 (dashed lines) films in RGB channels. Shaded bands represent the experimental uncertainties. (b): Change of the optical density relative to the value at 0.5 h post-irradiation. Irradiation was performed with a 6 MV photon beam at a dose of 2 Gy.

The film darkening behavior showed asymptotic behavior after approximately 40 h, with only minor subsequent changes, in agreement with Khan *et al.* [1].

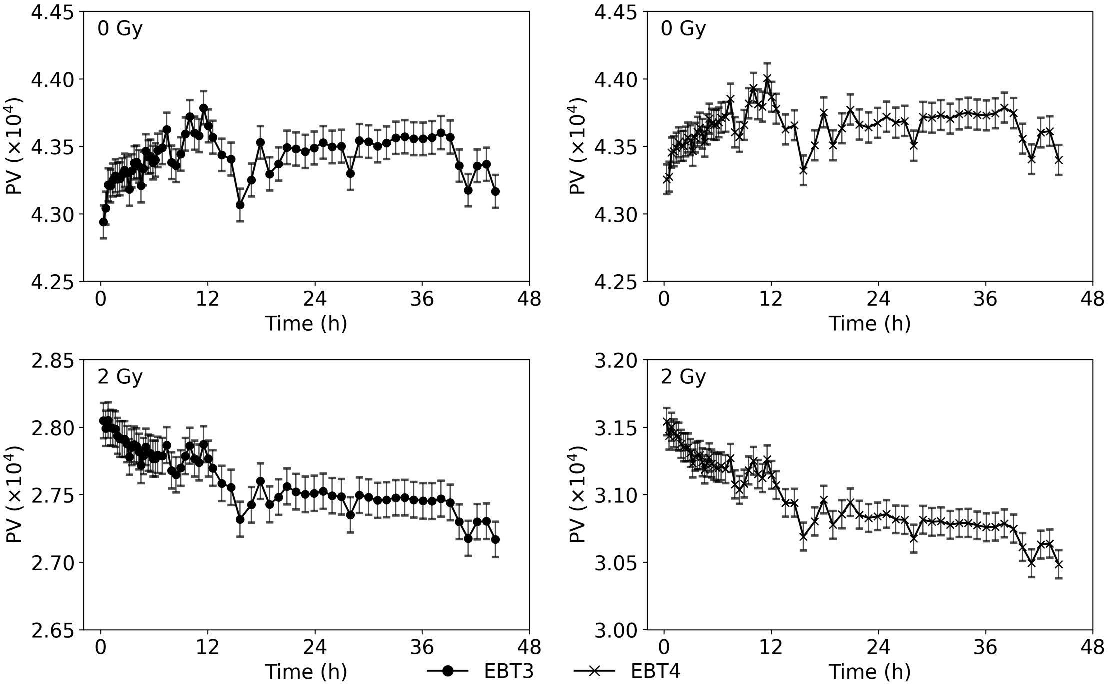

Figure S2: Film darkening reported in change of pixel values for EBT3 (circle) and EBT4 (crosses) films. The upper row represents non-irradiated films (i.e. 0 Gy) and the lower row films irradiated with 2 Gy.

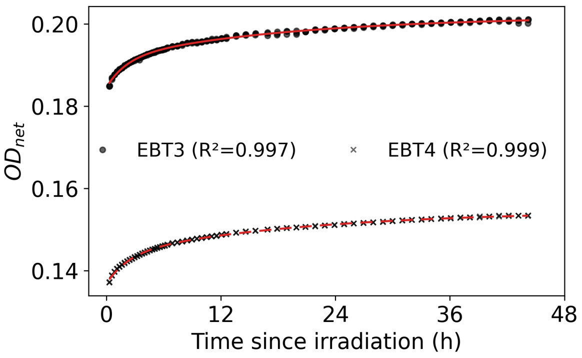

Figure S3: Applying the bi-exponential function proposed by Khan *et al.* to our experimental data.

Table S1: Bi-exponential darkening model parameters for EBT3 and EBT4 films (red channel at 2 Gy).

Parameter	EBT3	EBT4
C_1	0.00698 ± 0.00021	0.00703 ± 0.00015
T_1 (h)	2.10 ± 0.12	2.30 ± 0.09
C_2	0.01039 ± 0.00013	0.01079 ± 0.00009
T_2 (h)	19.24 ± 1.06	20.55 ± 0.81

Supplementary Material B

To provide information on the scan-to-scan variability of EBT3 and EBT4 films, we followed the methodology proposed by Lewis and Devic [2]. Consecutive scans of four film pieces irradiated to different dose levels (0, 0.5, 2 and 5 Gy) were acquired at time intervals of 2 min. The films had been irradiated approximately 1 yr prior to the measurements, thus any influence of post-exposure darkening can be considered negligible. Consequently, the observed variations can be attributed primarily to the scanner. For each film piece, the average pixel value was determined from a centered $2 \times 2\,\mathrm{cm}^2$ region of interest (ROI). The pixel values were normalized to the first scan for each dose level and film type. As shown in Figure S4, the mean deviation for EBT3 ranged between 0.1 to 0.3 %, whereas for EBT4 it ranged from 0.3 to 0.6 %. The maximum observed difference between two scans was 1.4 % for EBT3 and 0.8 % for EBT4, both occuring at at 5 Gy. As highlighted by Lewis and Devic [2], scan-to-scan variations of up to 1 % in the measured pixel value can translate into dose differences of several percent, depending on the local slope of the calibration curve.

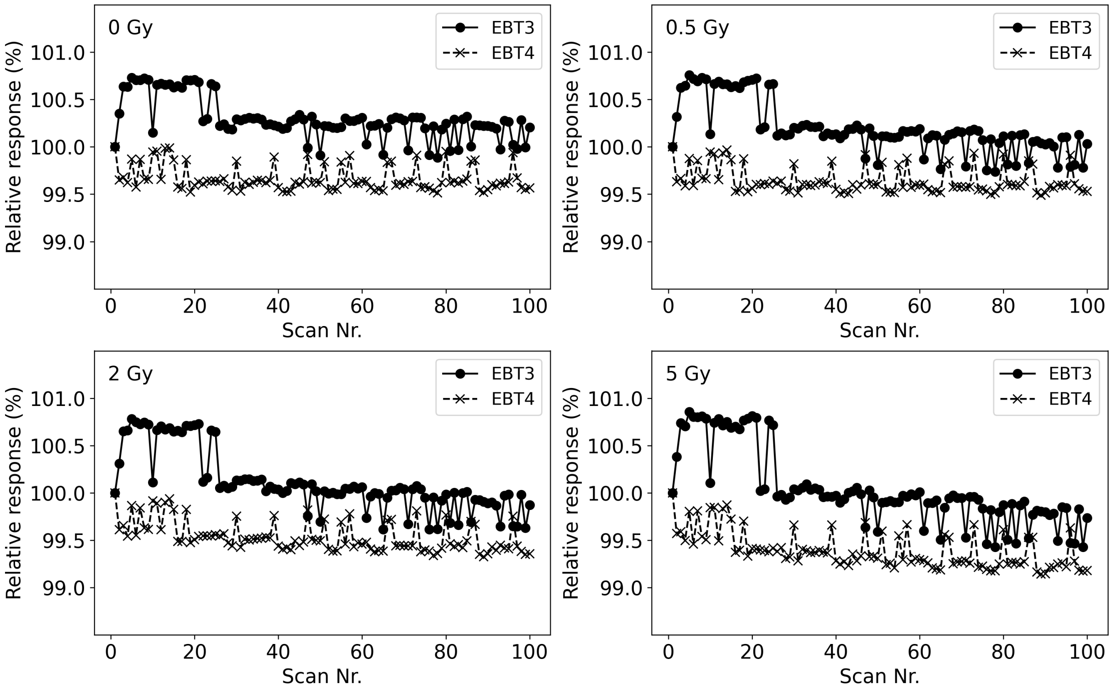

Figure S4: Relative response (red color channel) for 100 scans of EBT3 and EBT4 films at 2 min intervals.

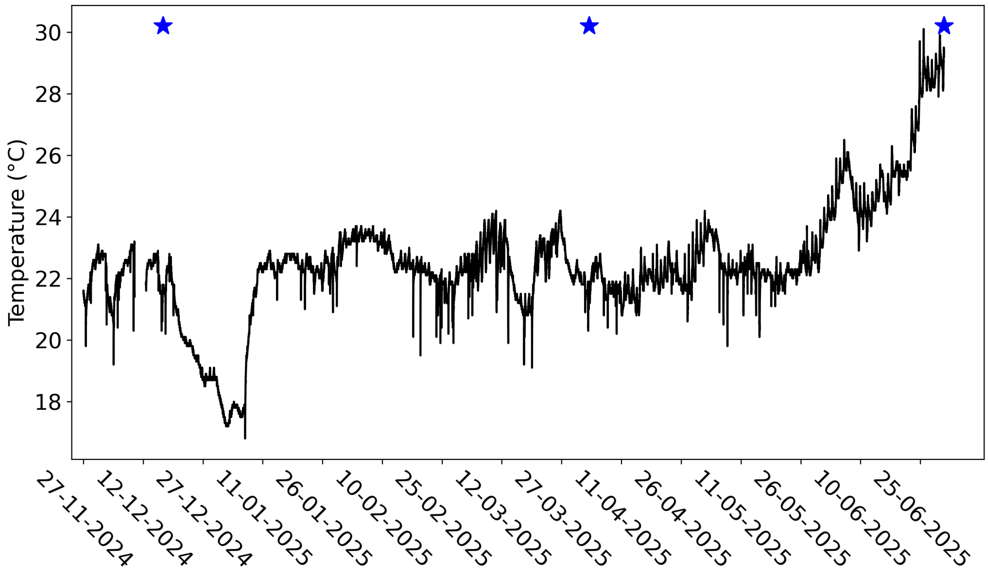

Figure S5: Temperature profile of storage location C (not temperature controlled) over the observation period, starting from the date of film calibration. Measurements were taken 20, 127, and 216 days thereafter (indicated by blue stars).

Table S2: SNR(PV) comparison of EBT3 and EBT4 at different dose levels for photon and proton irradiation using the **green** and **blue** channels. Ratios are relative difference (EBT4 vs. EBT3). Bold values imply statistically significant differences ($p < 0.05$), with EBT4 performing significantly better than EBT3.

Dose	**Green channel** — SNR(PV)						**Blue channel** — SNR(PV)					
(Gy)	Photon			Proton			Photon			Proton		
	EBT3	EBT4	Δ(%)	EBT3	EBT4	Δ(%)	EBT3	EBT4	Δ(%)	EBT3	EBT4	Δ(%)
0.2	234±13	231±6	-1	236±11	209±8	-12	186±8	189±8	2	188±12	181±9	-4
0.5	212±10	223±6	5	216±5	204±9	-6	179±7	194±5	**9**	185±8	174±7	-6
1.0	203±9	203±19	0	206±5	199±7	-3	184±9	178±11	-3	186±7	179±5	-4
1.5	186±4	195±13	5	197±4	204±6	4	175±3	177±13	1	185±2	185±12	0
2.0	177±7	196±2	**11**	183±5	192±6	5	171±9	182±5	6	179±6	180±7	1
2.5	167±2	181±5	**8**	162±9	184±2	**13**	169±3	173±9	2	168±6	177±5	5
3.0	154±9	177±7	**15**	158±7	173±10	9	161±7	180±6	**12**	163±7	169±11	4
5.0	125±5	145±10	**16**	131±2	151±5	**15**	147±6	157±14	7	151±4	163±8	8
8.0	99±1	121±2	**22**	99±3	120±3	**22**	139±2	152±3	**9**	133±6	148±2	**12**
10	84±2	104±2	**24**	81±3	106±4	**30**	124±3	138±3	**11**	119±12	135±6	14

Table S3: SNR(Dose) comparison of EBT3 and EBT4 at different dose levels for photon and proton irradiation using the **green** and **triple** channel approach. Ratios are given as relative difference (EBT4 vs. EBT3). Bold values imply statistically significant differences ($p < 0.05$), with EBT4 performing significantly better than EBT3.

Dose	**Green channel** — SNR(Dose)						**Triple channel** — SNR(Dose)					
(Gy)	Photon			Proton			Photon			Proton		
	EBT3	EBT4	Δ(%)	EBT3	EBT4	Δ(%)	EBT3	EBT4	Δ(%)	EBT3	EBT4	Δ(%)
0.2	10±0	7±1	-26	10±1	6±0	-44	8±0	6±0	-18	8±0	6±1	-29
0.5	18±4	12±0	-31	16±0	12±0	-25	16±1	14±2	-12	16±0	12±0	-25
1.0	33±1	23±3	-30	34±0	22±3	-35	32±4	25±1	-23	26±0	22±2	-16
1.5	38±0	29±2	-25	44±7	31±2	-30	39±0	32±3	-17	35±3	30±1	-15
2.0	49±3	40±0	-20	50±0	38±3	-23	35±3	39±3	**12**	35±3	35±3	-1
2.5	49±1	42±1	-16	48±3	42±0	-12	35±1	41±0	**19**	35±2	39±3	13
3.0	49±3	50±1	3	50±1	46±4	-8	31±2	42±0	**35**	31±2	41±2	**32**
5.0	49±2	54±2	**12**	50±1	56±2	**12**	25±1	43±2	**70**	25±1	44±2	**73**
8.0	47±0	59±2	**25**	46±1	63±2	**36**	23±0	42±1	**87**	22±1	44±0	**99**
10	44±1	58±2	**30**	43±2	65±2	**50**	23±0	42±0	**80**	22±0	43±0	**97**

Table S4: Uncertainty budget of reference measurements with the PTW 34001 Roos in protons. All reported uncertainties are expressed with a coverage factor $k = 1$. Uncertainty No. 4 and No. 5 are based on values provided in Table 38 of the TRS398-rev1 [3].

No.	Quantity or source of uncertainty	Uncertainty type	Probability density function	rel. standard uncertainty (%)
1	Repeatability, M_Q	A	Normal	0.25
2	$N_{D,w}$ reference chamber	B	Normal	0.7
3	Cross calibration (PTW 34001 Roos)	B	Normal	0.1
4	Correction for influence quantities k_i	B	Normal	0.3
5	Beam quality correction, k_Q	B	Normal	1.4
			**Combined uncertainty**	1.6

Table S5: Uncertainty budget of the film storage measurements. All reported uncertainties are expressed with a coverage factor $k = 1$. Uncertainty No. 1 is expressed as a range, reflecting its dependence on the applied dose level.

No.	Quantity or source of uncertainty	Uncertainty type	Probability density function	rel. standard uncertainty (%)
1	Readout of film area	A	Normal	0.4–1.0
2	Setup positioning uncertainty	B	Normal	0.1
3	Correction of influence quantities	B	Normal	0.1
4	Long-term stability of ionization chamber	B	Normal	0.1
5	LINAC output stability	B	Normal	0.2
			**Combined uncertainty**	0.5–1.0

References

[1] Khan, R., Rahimi, R., Fan, J., Chen, K.L.. Systematic characterization of new EBT4 radiochromic films in clinical x-ray beams. Biomed Phys Eng Express 2024;11:015006. doi:10.1088/2057-1976/ad8c49.

[2] Lewis, D., Devic, S.. Correcting scan-to-scan response variability for a radiochromic film-based reference dosimetry system. Med Phys 2015;42(10):5692–701. doi:10.1118/1.4929563.

[3] Absorbed Dose Determination in External Beam Radiotherapy. No. 398 (Rev. 1) in Technical Reports Series; International Atomic Energy Agency; 2024. ISBN 9789201460226. doi:10.61092/iaea.ve7q-y94k.
